# METTL3 blocked the progression of diabetic retinopathy through m6A-modified SOX2

**DOI:** 10.1515/med-2025-1191

**Published:** 2025-07-25

**Authors:** Xiujuan Chen, Qipeng Ling, Jie Xu, Yunyao Ye, Lili Dong

**Affiliations:** Ophthalmology Department, The Affiliated Taizhou People’s Hospital of Nanjing Medical University, Taizhou School of Clinical Medicine, Nanjing Medical University, Taizhou, 225300, China; Institute of Clinical Medicine, The Affiliated Taizhou People’s Hospital of Nanjing Medical University, Taizhou School of Clinical Medicine, Nanjing Medical University, Taizhou, 225300, China; Department of Oncology, The Affiliated Taizhou People’s Hospital of Nanjing Medical University, Taizhou School of Clinical Medicine, Nanjing Medical University, Taizhou, 225300, China

**Keywords:** diabetic retinopathy, METTL3, SOX2

## Abstract

We aimed to explore the regulatory effects of methyltransferase-like 3 (METTL3) on diabetic retinopathy (DR) by regulating the m6A modification of *SOX2* mRNA and elucidating the underlying molecular mechanism. The DR model was established by stimulating human retinal endothelial cells (HRECs) with high glucose (HG). METTL3, insulin-like growth factor 2 binding protein 2 (IGF2BP2), and SOX2 levels in the sera of patients with DR and HRECs were determined using qRT-PCR and western blotting. Moreover, the interactions between SOX2 and METTL3 or IGF2BP2 were confirmed using RNA-binding protein immunoprecipitation (RIP) experiments. Furthermore, HRECs proliferation and apoptosis were determined using 3-(4,5-dimethylthiazol-2-yl)-2,5-diphenyltetrazolium bromide (MTT) and flow cytometry, respectively. The protein level of cleaved-caspase3 and caspase3 in HRECs were evaluated using western blotting. The results indicated that the expression of METTL3, IGF2BP2, and SOX2 was notably decreased in the serum of patients with DR, as well as in HRECs under HGs. RIP further verified the relationship between *METTL3* and *SOX2* mRNA expression. HG treatment inhibited HREC viability, increased apoptosis, and enhanced cleaved-caspase3 expression and cleaved-caspase3/caspase3 ratio. Upregulation of *METTL3* significantly restored the effects of HG, whereas *SOX2* knockdown partially reversed the regulatory effects of METTL3 on HRECs. In summary, METTL3 blocks the progression of DR by regulating m6A modification on *SOX2* mRNA.

## 1 Introduction

Diabetic retinopathy (DR), a major complication of diabetes [1], can cause irreversible visual impairment and even result in blindness [2]. Approximately 80% of the patients with diabetes eventually develop DR [3]. Retinal inflammation, retinal vascular permeability, and the retinal barrier are involved in the pathological progression of DR [4,5]. Currently, laser photocoagulation, anti-vascular endothelial growth factor drugs, and glucocorticoid therapy are the main therapies for DR; however, these treatments have side effects [6,7]. Abnormal endothelial cell function caused by hyperglycemia is considered the primary cause of DR [8]. Currently, high glucose (HG)-induced human retinal endothelial cells (HRECs) or hRMECs damage has been used to establish DR models *in vitro* to illustrate the roles of regulated proteins in the progression of DR. Zhang et al. [9] revealed that CKIP-1 regulates cell viability, oxidative stress, inflammation, and apoptosis in HG-stimulated HRECs through the Nrf2/ARE signaling pathway. Liu et al. [10] suggested that the *ZFAS1* lncRNA positively facilitates endothelial ferroptosis via the miR-7-5p/ACSL4 axis in HRECs. Thus, understanding the regulatory mechanisms of HRECs under HG conditions is of great significance for the progression of DR.

N6-methyladenosine (m6A), one of the most common mRNA modifications in eukaryotes, is widely used to regulate gene expression [11]. M6A plays a wide range of roles in early embryonic development, obstetric syndromes, tumorigenesis, and diabetes mellitus [12,13,14,15]. METTL3, methyltransferase-like 14, and its cofactor Wilms’ tumor-1 associated protein determine the m6A methyltransferase complex that catalyzes m6A modification [16]. METTL3 maintains m6A methylation homeostasis by methylating target mRNA and is involved in a variety of pathological processes, including colorectal carcinoma [17], breast cancer [18], chronic obstructive pulmonary disease [19], and DR [20]. Furthermore, Cao et al. [21] suggested that METTL3 regulates the endothelial–mesenchymal transition in DR through the SNHG7/KHSRP/MKL1 lncRNA axis. Studies have also revealed the decrease of METTL3 in DR [21,22,23]. Previous studies have confirmed a relationship between METTL3 and SOX2. Xie et al. [24] revealed that circVMP1 acts as a miR-524-5p sponge to upregulate METTL3 and SOX2 expression in non-small-cell lung cancer. Wang and Yang [25] demonstrated that METTL3 relieved SH-SY5Y cell injury in postoperative cognitive dysfunction by regulating m6A and mRNA levels of *SOX2*. However, whether METTL3 regulates the m6A modification of SOX2 in DR remains unclear.

Insulin-like growth factor 2 binding protein 2 (IGF2BP2), one of the members of the IGF2BP family, recognizes m^6^A-modified mRNAs to regulate the progression of diseases [26]. IGF2BP2 regulates disease progression, including acute myeloid leukemia [27] and colorectal cancer [28,29]. IGF2BP2 is also a potential target for the treatment of DN. In addition, Yi et al. [30] found that the METTL3/IGF2BP2 axis influences colorectal cancer development by mediating m6A modification of STAG3. Xie et al. [31] confirmed the binding between *METTL3* and *SOX2* mRNA, and the silencing of METTL3 reduced the enrichment of SOX2 in anti-IGF2BP2 antibodies in breast cancer. However, the exact function and mechanism of the METTL3/IGF2BP2 axis in the regulation of DR progression remain unclear.

Based on these findings, we speculate that METTL3 promotes DR progression through the m6A methylation of *SOX2* mRNA via IGF2BP2-dependent mechanisms. Our results provide novel insights into METTL3-mediated m6A modifications for clinical monitoring and therapies for DR.

## 2 Materials and methods

### 2.1 Clinical sample collection

Peripheral venous blood samples were aseptically collected from the patients with DR (DR group, *n* = 30; 15 males, 15 females, age from 31 to 65 years) and healthy volunteers (control group, *n* = 30; 15 males, 15 females, age from 29 to 67 years) at the Affiliated Taizhou People’s Hospital of Nanjing Medical University. Then, samples were aliquoted and stored at −80°C until further analysis. All samples were labeled with group and subject information for accurate identification and subsequent processing. All participants signed an informed consent form before using their clinical samples. The use of samples for this study was approved by the Ethics Committee of the Affiliated Taizhou People’s Hospital of Nanjing Medical University.

### 2.2 Cell culture and treatment

HRECs were purchased from American Type Culture Collection (Manassas, VA, USA) and maintained in dulbecco's modified eagle medium (DMEM) medium (Gibco, Carlsbad, CA, USA) with 10% fetal bovine serum (Gibco, USA) and 100 U/mL penicillin/streptomycin (Gibco), kept in the incubator at 37°C with 5% CO_2_. To establish the DR model *in vitro*, HRECs were incubated in normal glucose (5.5 mmol/L) or HG (30 mmol/L) with DMEM medium for 48 h in the incubator at 37°C with 5% CO_2_ [21,22,23,32].

### 2.3 3-(4,5-dimethylthiazol-2-yl)-2,5-diphenyltetrazolium bromide (MTT) assay

After being transfected for 48 h, HRECs were seeded in 96-well plates and incubated for 24 h at 37°C. Then, cells were treated with 10 μL MTT solutions (M1025, Solarbio) and continuously incubated for further 4 h. After treatment, the solution was discarded and 100 μL DMSO (D8371, Solarbio) was added to degrade the formazan product. Finally, the optical density at the wavelength of 490 nm was determined using a microplate reader (BioTek, Richmond, USA) after 15 min of vibration mixing, following the manufacturer’s protocol.

### 2.4 Flow cytometry

HRECs were cultured in DMEM until they reached 80% confluence. The cells were digested with trypsin and washed with PBS. HRECs were washed and stained using an Annexin V-FITC Apoptosis Detection Kit (C1062S, Beyotime, China) at room temperature for 15 min in the dark, according to the manufacturer’s protocol. The Kaluza Analysis software was used to quantify the number of apoptotic cells.

### 2.5 Cell transfection

HRECs were cultured in DMEM until they reached 80% confluence. The control-siRNA, *SOX2*-siRNA, control-plasmid, *IGF2BP2*-plasmid, and METTL3-plasmid were transfected into HRECs using the Lipofectamine 3,000 reagent (L3000001; Thermo Fisher Scientific, Waltham, MA, USA) following the manufacturer’s instructions. After 48 h of transfection, qRT-PCR and western blotting were performed to assess transfection efficiency.

### 2.6 qRT-PCR analysis

Total RNA was extracted from HRECs and blood samples by TRIpure Total RNA Extraction Reagent (EP013, ELK Biotechnology) according to the manufacturer’s instructions and was reverse-transcribed into cDNA by a PrimeScript™ 1st Strand cDNA Synthesis Kit (6210B, TaKaRa, Japan). Then, the EnTurbo™ SYBR Green PCR SuperMix (EQ001, ELK Biotechnology) with QuantStudio™ 5 Real-Time PCR System (Thermo Fisher Scientific) was utilized for qRT-PCR, and the reactions were carried out with the following cycling conditions: 40 cycles for 15 min at 95°C, 10 s at 95°C, 30 s at 60°C, 30 s at 72°C, 60 s at 95°C and 11 min at 55°C. Subsequently, the relative mRNA expression levels were calculated using the 2^−ΔΔCt^ method, and glyceraldehyde 3-phosphate dehydrogenase was regarded as a control.

### 2.7 Western blotting

Total protein from HRECs and blood samples was extracted using radio immunoprecipitation assay buffer (AS1004, ASPEN), and the supernatants were collected. After the protein concentration was determined using the BCA Protein Quantification Kit (P0010, Thermo Fisher Scientific). Equal amounts of protein were mixed with SDS buffer, boiled for 5 min, separated using 10% SDS-PAGE, and transferred onto polyvinylidene difluoride membranes (Millipore). Subsequently, the membranes were blocked with 5% skimmed milk in tris buffered saline with Tween 20 (TBST) and cultivated overnight at 4°C with primary antibodies against METTL3 (15073-1-AP, 1:1,000, Wuhan Sanying), IGF2BP2 (ab124930, 1:1,000, Abcam), SOX2 (#23064, 1:1,000, CST), cleaved-Caspase3 (AF7022, 1:500, affbiotech), Caspase3 (AF6311, 1:5,000, affbiotech), and β-actin (TDY051, 1:1,000, Beijing TDY Biotech CO., LTD.). After washing three times with TBST, the membranes were incubated with Goat Anti-Rabbit IgG HRP antibody (AS1107, ASPEN, 1:5,000) and detected by the Novex™ ECL Chemiluminescent Substrate Reagent Kit (WP20005, Thermo Fisher Scientific). Relative protein expression was detected using the ImageJ software (Bethesda, MD, USA).

### 2.8 RNA-binding protein immunoprecipitation (RIP) assay

RIP assay was conducted according to the directions of Magna RIP^®^RNA Binding Protein immunoprecipitation Kit (17-700, Merck Millipore, Germany). Briefly, the cleavage products were obtained from HRECs using RIP lysis buffer containing RNase inhibitors. Then, anti-METTL3 (ab195352, 1:50, Abcam), anti-IGF2BP2 (ab313422, 1:100, Abcam), or anti-IgG was mixed with Protein A and incubated with cell lysate at 4°C overnight. Subsequently, the proteins were digested with proteinase K, and the RNAs were purified. Finally, the immunoprecipitated RNAs were subjected to qRT-PCR to assess their relative levels.

### 2.9 MeRIP-qPCR analysis

The Magna meRIP M6A Kit (Millipore, Germany) was used to apply the meRIP assay for the purpose of determining m6A enrichment according to the manufacturer’s protocols. Total RNA of HRECs was extracted. The RNA samples were subjected to immunoprecipitation with magnetic beads containing either anti-m6A antibody or anti-IgG. The RNAs that were coprecipitated were purified and then dissolved in RNAse-free water. RT‒qPCR was used to analyze m6A enrichment of binding RNA targets.

### 2.10 Statistical analysis

All experiments were independently repeated three times. Data are presented as means ± standard deviation. Comparisons between two groups were assessed using Student’s *t*-test, and differences among multiple groups were analyzed using one-way analysis of variance followed by Tukey’s post hoc test. All experiments were conducted in triplicates. All statistical analyses were performed using GraphPad Prism 8.0 software. Statistical significance was set at *P* < 0.05.


**Ethics approval and consent to participate:** All subjects signed informed consent before using clinical samples. This study has been approved by the ethics committee of The Affiliated Taizhou People’s Hospital of Nanjing Medical University.
**Consent for publication:** All patients agreed to publication.

## 3 Results

### 3.1 METTL3, IGF2BP2, and SOX2 were down-regulated in the serum of patients with DR

**Figure 1:**
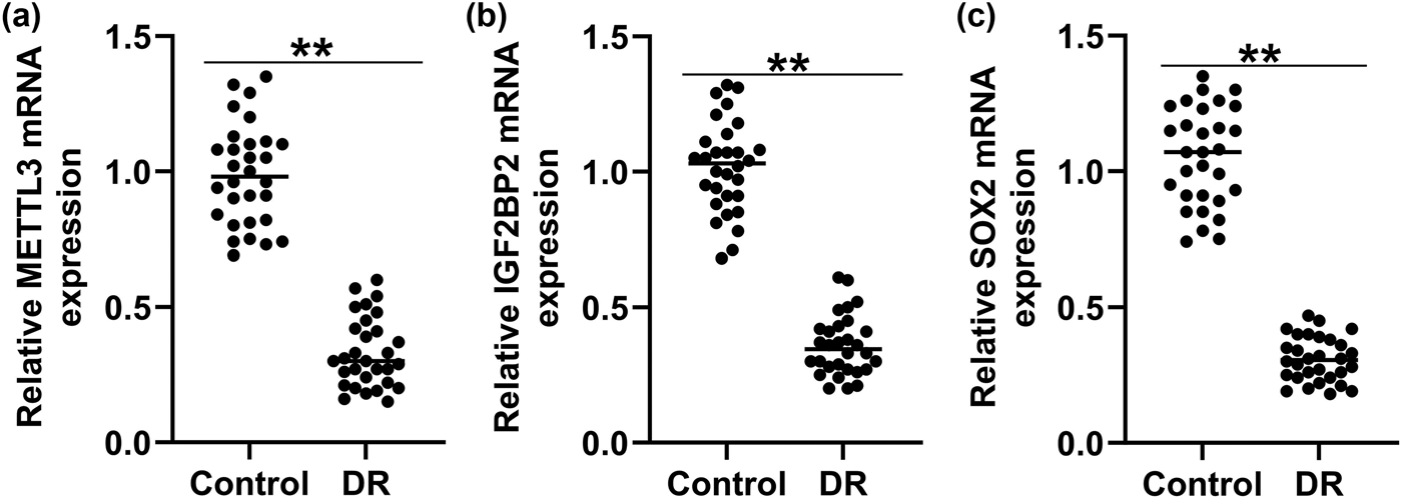
METTL3, IGF2BP2, and SOX2 expression in serum of patients with DR. *METTL3* (a), *IGF2BP2* (b), and *SOX2* (c) mRNA levels were assessed using qRT-PCR. Values are presented as mean ± SD. *N* = 3. **P < 0.01.

To confirm the roles of METTL3, IGF2BP2, and SOX2 in the progression of DR, the levels of METTL3, IGF2BP2, and SOX2 in the serum of patients with DR were determined using qRT-PCR. As presented in Figure 1a–c, METTL3, IGF2BP2, and SOX2 levels in the serum of patients with DR were markedly lower than those in healthy volunteers. Our data demonstrated that METTL3, IGF2BP2, and SOX2 are vital regulators of DR progression.

### 3.2 METTL3, IGF2BP2, and SOX2 expression were suppressed in HG-induced HRMEC

**Figure 2:**
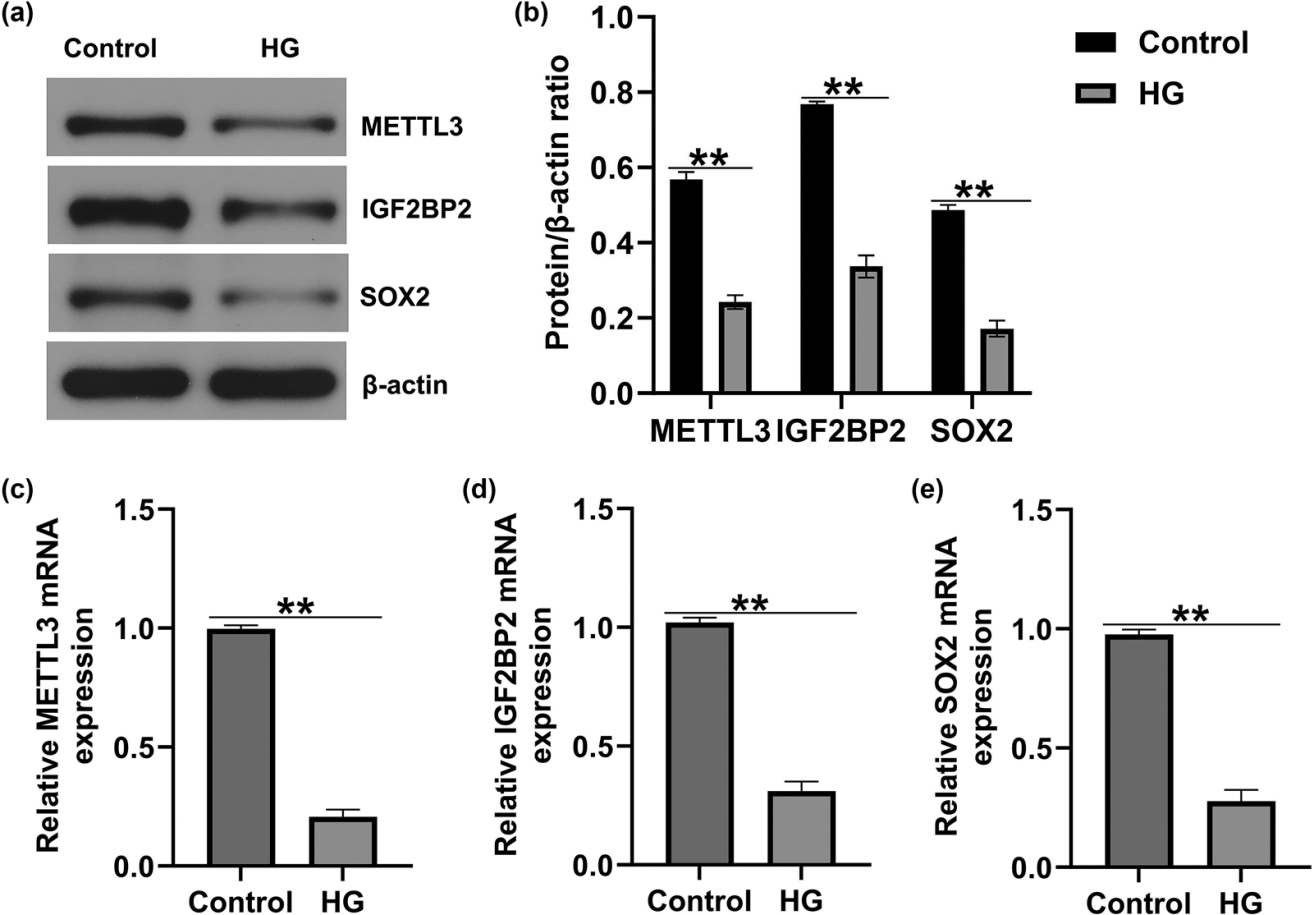
METTL3, IGF2BP2, and SOX2 expression in HG-treated HRECs. (a) and (b) Detection of METTL3, IGF2BP2 and SOX2 expression in HG-stimulated HRECs using western blotting. *METTL3* (c), *IGF2BP2* (d), and *SOX2* (e) mRNA levels were measured using qRT-PCR in HG-stimulated HRECs. Data are presented as mean ± SD. *N* = 3. **P < 0.01.

We determined the expression levels of METTL3, IGF2BP2, and SOX2 in the HG-induced DR model *in vitro*. In contrast to the control group, the HG group showed a notable decrease in METTL3, IGF2BP2, and SOX2 protein levels in HRECs (Figure 2a and b). In addition, the mRNA expression of *METTL3*, *IGF2BP2*, and *SOX2* was significantly inhibited in HRECs exposed to HG (Figure 2c–e), indicating that *METTL3* affects DR under HG conditions via the IGF2BP2/SOX2 axis.

### 3.3 METTL3 induced m6A methylation on SOX2

**Figure 3:**
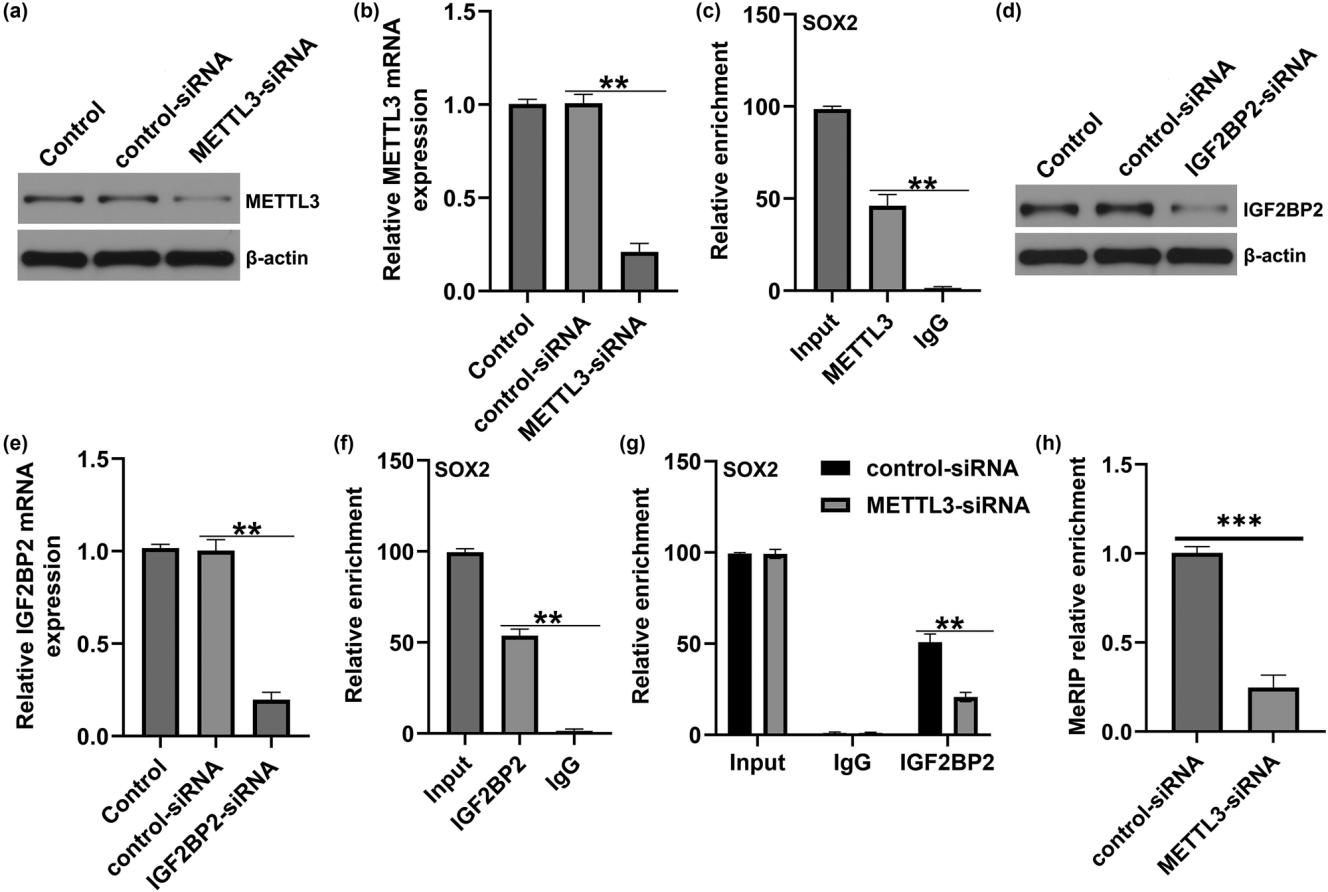
METTL3 induces m6A modification on SOX2. (a) Protein expression of METTL3 in HRECs transfected with control-siRNA or METTL3-siRNA. (b) mRNA level of METTL3 in control-siRNA or METTL3-siRNA-transfected HRECs. (c) Relative enrichment of SOX2 in input, anti-METTL3, and anti-IgG. (d) Expression of IGF2BP2 in HRECs transfected with control-siRNA or *IGF2BP2*-siRNA was determined by western blot. (e) RT-qPCR analysis of *IGF2BP2* mRNA level in control-siRNA or *IGF2BP2-*siRNA-transfected cells. (f) Relative enrichment of SOX2 in input, anti-IGF2BP2, and anti-IgG after transfection of control-plasmid or METTL3-plasmid in HRECs. (g) Relative enrichment of SOX2 in input, anti-IGF2BP2, and anti-IgG after transfection of control-siRNA or METTL3-siRNA in HRECs. (h) Enrichment of METTL3-mediated SOX2 m6A modification using meRIP-qPCR. Results are displayed as mean ± SD. *N* = 3. **P < 0.01; ***P < 0.001.

We then revealed the relationship between METTL3 and SOX2 in the development of DR. *METTL3*-siRNA was used to repress METTL3 expression in HRECs, and control siRNA or METTL3-siRNA was transfected into HRECs. The results of RT-qPCR and western blotting suggested that METTL3 was down-regulated in METTL3-siRNA-treated HRECs (Figure 3a and b). Furthermore, RIP assay results indicated that compared with the anti-IgG group, the relative enrichment of SOX2 was significantly enhanced in the anti-METTL3 group, suggesting an interaction between METTL3 and SOX2 (Figure 3c). Similarly, RT-qPCR and western blotting assays revealed that IGF2BP2 levels significantly decreased after IGF2BP2-siRNA treatment (Figure 3d and e). RIP assay revealed that compared with the anti-IgG group, the relative enrichment of SOX2 was significantly enhanced in the anti-IGF2BP2 group, suggesting the interaction between IGF2BP2 and SOX2 (Figure 3f), and their interaction ability was reduced after METTL3 downregulation (Figure 3g). To validate the potential impact of METTL3-mediated m6A modification on SOX2, we conducted meRIP-PCR analysis, which indicated that the m6A level of SOX2 decreased significantly after METTL3 was down-regulated (Figure 3h). Our findings revealed that METTL3 regulates SOX2 levels via m6A methylation of *SOX2* mRNA.

### 3.4 Transfection efficiency of METTL3-plasmid and *SOX2*-siRNA in HRECs

**Figure 4:**
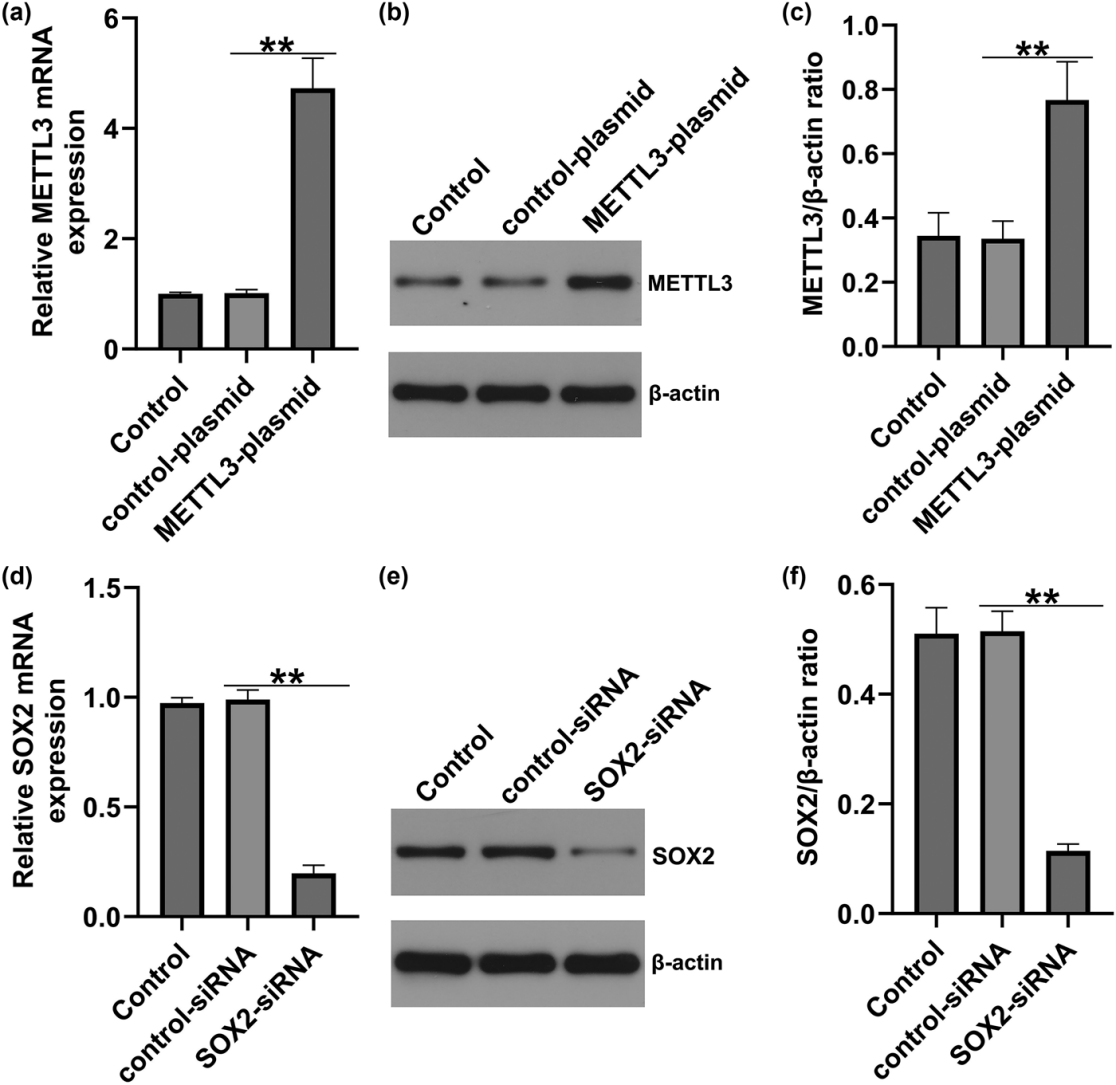
Expressions of METTL3 and SOX2 in METTL3-plasmid or SOX2-siRNA-transfected HRECs. HRECs were transfected with control-plasmid, METTL3-plasmid, control-siRNA, or SOX2-siRNA for 48 h. (a) Relative levels of METTL3 were detected by qRT-PCR. (b) and (c) Protein expression of METTL3 was measured by western blotting. (d) Relative SOX2 levels were detected by qRT-PCR. (e) and (f) Protein expression of SOX2 was measured by western blotting. Results are displayed as mean ± SD. *N* = 3. **P < 0.01.

To further investigate whether METTL3 regulates the HREC function by regulating SOX2, the control plasmid, METTL3 plasmid, control siRNA, and SOX2-siRNA were transfected into HRECs. qRT-PCR and western blotting were used to evaluate transfection efficiency. We found that both the mRNA and protein levels of METTL3 were notably increased by METTL3-plasmid (Figure 4a–c). Compared with the control-siRNA group, the mRNA and protein levels of SOX2 were significantly reduced in SOX2-siRNA-transfected HRECs (Figure 4d–f).

### 3.5 SOX2-siRNA reversed the effects of METTL3-plasmid on METTL3 and SOX2 expression

**Figure 5:**
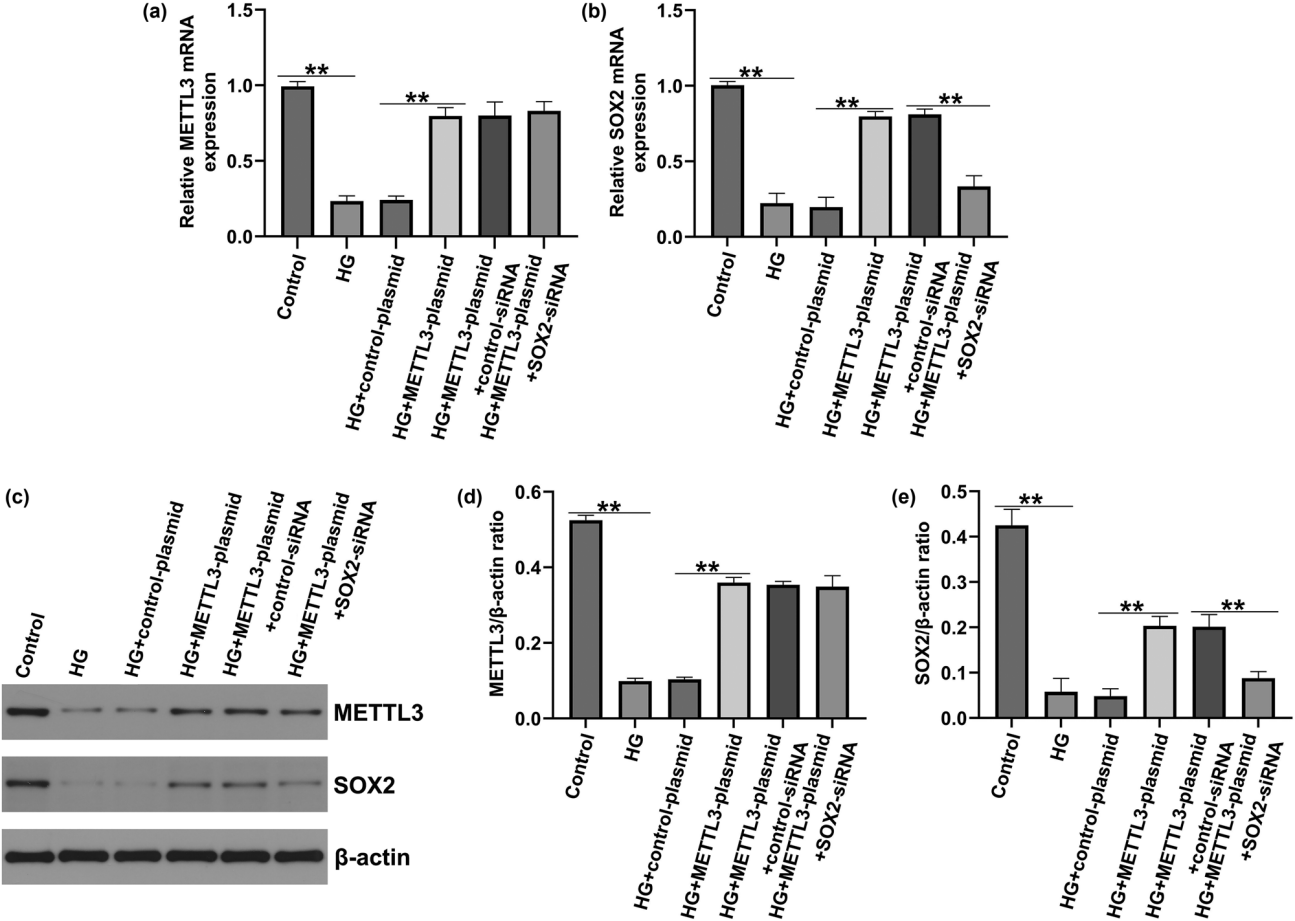
METTL3-plasmid or SOX2-siRNA affected the effects of HG treatment on the expressions of METTL3 and SOX2 in HRECs. HRECs were pre-transfected with control plasmid, METTL3-plasmid, control siRNA, or *SOX2*-siRNA for 2 h, and then exposed to 30 mmo/L HG for 48 h. The cells were divided into six groups: Control, HG, HG + control-plasmid, HG + METTL3-plasmid, HG + METTL3-plasmid + control-siRNA, and HG + METTL3-plasmid + *SOX2*-siRNA group. (a) and (b) Relative levels of METTL3 and SOX2 in the groups were evaluated using qRT-PCR. (c)–(e) Western blotting of METTL3 and SOX2 expression. Values are represented as mean ± SD. *N* = 3. *P < 0.05, **P < 0.01 vs control.

To elucidate the regulatory roles of METTL3 and SOX2 in DR, HRECs were stimulated with HG and transfected with control-siRNA, SOX2-siRNA, control-plasmid, and METTL3-plasmid. As presented in Figure 5a and b, compared to the control group, HG led to reduced METTL3 mRNA levels and protein expression, while the additional METTL3-plasmid enhanced this effect. Furthermore, we observed reduced METTL3 and SOX2 protein expression in HG-stimulated HRECs compared with those in the control group (Figure 5c–e), and this reduction was reversed by METTL3-plasmid. Compared to the HG + METTL3-plasmid + control-siRNA group, SOX2 was significantly down-regulated in HG + METTL3-plasmid + SOX2-siRNA-treated HRECs. Our results demonstrated that SOX2 is involved in METTL3-mediated HRECs.

### 3.6 METTL3 regulates HG-induced HREC viability and apoptosis via m6A-modified SOX2 in DR.

**Figure 6:**
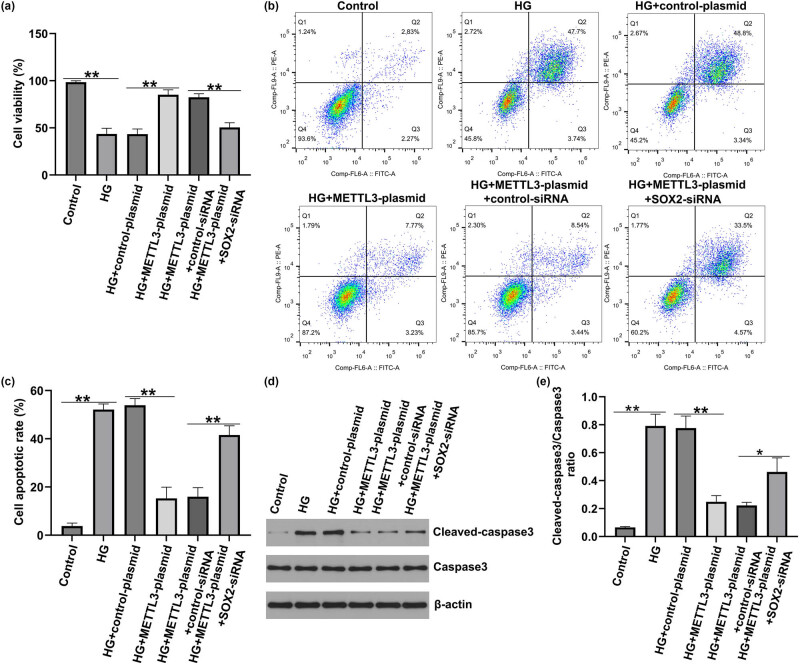
Effect of SOX2-siRNA and METTL3-plasmid on HG-induced HRECs viability and apoptosis. HRECs were re-transfected with control plasmid, METTL3-plasmid, control siRNA, or SOX2-siRNA for 2 h, and then exposed to 30 mmo/L HG for 48 h. The cells were divided into six groups: Control, HG, HG + control-plasmid, HG + METTL3-plasmid, HG + METTL3-plasmid + control-siRNA, and HG + METTL3-plasmid + SOX2-siRNA group. (a) MTT assay for HREC viability. (b). Analysis of apoptosis in HRECs was performed using flow cytometry. (c) Quantification of apoptotic HRECs. (d) Western blotting detection of cleaved-caspase3 and caspase3 expression in HRECs. (e) Analysis of cleaved-caspase3/caspase3 ratio. Results are displayed as mean ± SD. *P < 0.05, **P < 0.01.

Finally, we assessed whether METTL3 regulated HREC viability and apoptosis by regulating SOX2 expression. Under HG conditions, HREC viability was reduced (Figure 6a), apoptosis was increased (Figure 6b and c), cleaved-caspase3 expression was enhanced (Figure 6d), and cleaved-caspase3/caspase3 was elevated (Figure 6e). Moreover, upregulation of METTL3 increased cell viability and suppressed apoptosis, while *SOX2*-siRNA counteracted the effects of the METTL3 plasmid on cell viability and apoptosis. Western blotting indicated that the upregulation of METTL3 significantly decreased cleaved-caspase3 expression and the cleaved-caspase3/caspase3 ratio. *SOX2-*siRNA reversed the regulatory influence of METTL3-siRNA on apoptosis-related proteins. These observations indicate that METTL3 regulates HG-induced HREC viability and apoptosis via m6A-modified SOX2 in DR.

## 4 Discussion

DR, a dietary complication, is characterized by the loss of periretinal cells and abnormal angiogenesis. HG is the main factor resulting in the occurrence of DR. Long-term exposure of retinal cells to HG environments leads to endothelial cell proliferation and migration, retinal detachment, and microvascular damage [33,34]. Although DR is highly prevalent, the exact mechanism underlying its pathogenesis remains unclear. Currently, only a few effective methods are available to treat DR in clinical practice. Therefore, exploring the regulatory mechanisms underlying DR and finding novel methods to delay its progression are urgently needed. An increasing number of reports have shown that HG-stimulated HRECs have a similar pathogenesis to DR; HRECs were treated with HG to mimic DR. For instance, Liu et al. demonstrated that the upregulation of SPRED2 suppresses EMT and endothelial injury in HRECs via the mitogen-activated protein kinases signaling pathway in DR [35]. Our study is consistent with the results of previous studies, suggesting that HRECs can be incubated in normal glucose or HG to generate a DR model *in vitro*. Our findings may help better understand the regulatory mechanisms of DR progression and provide support for future research.

M6A methylation, the most common chemical methylation in human mRNA, regulates RNA expression through splicing and translation [36]. Several studies have suggested that aberrant m6A modification is closely associated with the development of diseases, including DR [15,20,37]. m6A levels have been observed in various major exacerbating factors in the pathogenesis of DR, such as angiogenesis, inflammation, and oxidative stress. Additionally, various non-coding RNAs, such as circRNA, lncRNA, and microRNA, are also associated with DR, and m6A has been shown to influence all of these non-coding RNAs [15]. Zhou et al. indicated that YTHDC1 aggravates HG-induced RVECs injury via m6A modification of CDK6 in DR [37]. METTL3, a vital component of the m6A methyltransferase complex, is involved in multiple types of cancers [17,18,38]. A recent study found that METTL3 was expressed at low levels in patients with DR, DR mice, and HG-stimulated HRMECs [21]. In addition, a study by Zha et al. suggested that METTL3 was downregulated in peripheral venous blood samples of patients with DM [22]. On the contrary, Fu et al. indicated that HG stimulation or diabetic stress resulted in an elevation in the overall m6A level, as well as the expression level of METTL3 in the experimental models of DR [39]. Consistent with the results of other studies [21,22,23], we found that the expression of METTL3 in patients with DR and HG-treated HRECs was lower than that in the corresponding control groups, indicating that METTL3 acts as a key regulatory agent during DR progression; however, the specific mechanism of METTL3 in DR needs to be further explored.

METTL3 is related to disease progression via m6A modification of *SIRT1* [40], *STEAP2* [41], and *HMGA2* mRNA [42]. Xie et al. confirmed the potential interaction between METTL3 and SOX2, and the relationship between IGF2BP2 and SOX2 affected by METTL3 was confirmed using the RIP assay [31]. However, whether METTL3/IGF2BP2-mediated m6A modification of *SOX2* mRNA is involved in DR progression remains unknown. Our study is the first to report the regulatory role of the METTL3/IGF2BP2/SOX2 axis in DR. We observed that the levels of IGF2BP2 and SOX2 were lower in the serum of patients with DR and in HRECs. The RIP assay suggested that METTL3 could induce m6A modifications on *SOX2* mRNA. IGF2BP2 binds to m6A-modified *SOX2* mRNA, and the relationship between IGF2BP2 and SOX2 is regulated by METTL3. We further demonstrated that the upregulation of METTL3 increased SOX2 expression in HRECs. These findings confirm the regulatory role of the METTL3 and IGF2BP2/SOX2 axes in DR progression, which is similar to the results reported by Xie et al. [31].

Previous studies have reported that retinal cells exposed to HG conditions for a long time may develop a series of fundus diseases, including endothelial cell apoptosis, proliferation, and migration. Feng et al. revealed that *KCTD10* knockdown increases cell viability and inhibits apoptosis in DR [43]. Moreover, Yu et al. showed that GLI enhanced cell viability and reduced the number of apoptotic HRECs [44]. In this study, we further illustrated the possible molecular mechanisms underlying METTL3 regulation in HRECs treated with HG. Upregulation of METTL3 promoted HG-stimulated HRECs viability and reduced apoptosis. In addition, the METTL3-plasmid suppressed the expression of apoptosis execution factor cleaved-caspase3 and the cleaved-caspase3/caspase3 ratio, further confirming the ability of METTL3 to block HG-induced damage in HRECs. However, the transfection of cells with *SOX2*-siRNA notably reversed these results, indicating that METTL3 promotes the progression of DR through m6A-modified SOX2. Unlike our results, Fu et al.’s study showed that METTL3 deficiency relieved HG-induced oxidative stress damage and inflammation in Müller cells [39].

However, our study has some limitations. For instance, a dose–response and time-course analysis of HG treatment for HRECs to determine the optimal conditions for *in vitro* modeling of DR was not performed. Besides, potential off-target effects of the siRNAs and overexpression constructs are other limitations of this study. Besides this, our study only confirmed the role of METTL3 in HRECs. However, the function of METTL3 in animal models of DR has not yet been elucidated. Moreover, immunofluorescence staining of retinal tissue sections from DR patients and controls to visualize the localization and expression patterns of METTL3, IGF2BP2, and SOX2; detection of the tube formation and migration ability of HRECs; and the analysis of potential SOX2 target genes were not applied in this study. These studies will make our research more convincing. In the future, we will address these issues.

Taken together, METTL3 is downregulated in DR. METTL3 blocks the progression of DR via inhibiting HG-induced apoptosis of HRECs by regulating m6A modification on SOX2 mRNA via IGF2BP2-dependent mechanisms. Our findings revealed that m6A methylation of SOX2 induced by METTL3 may be a therapeutic target for DR therapy. The research will provide new directions and strategies for the diagnosis and treatment of DR.
